# Using the Person-Based Approach to optimise a digital intervention for the management of hypertension

**DOI:** 10.1371/journal.pone.0196868

**Published:** 2018-05-03

**Authors:** Katherine Bradbury, Katherine Morton, Rebecca Band, Anne van Woezik, Rebecca Grist, Richard J. McManus, Paul Little, Lucy Yardley

**Affiliations:** 1 University of Southampton, Academic unit of psychology, University of Southampton, Southampton, United Kingdom; 2 Rotterdam University of Applied Science, Healthcare technology, Rotterdam University of Applied Science, Rotterdam, Netherlands; 3 Brighton, School of Applied Social Science, Falmer, Brighton, United Kingdom; 4 University of Oxford, Nuffield Department of Primary Care Health Sciences, University of Oxford, Oxford, United Kingdom; 5 University of Southampton, Primary Care and Population Sciences, Faculty of Medicine, University of Southampton, Southampton, United Kingdom; The University of Sydney, AUSTRALIA

## Abstract

**Background:**

For behaviour-change interventions to be successful they must be acceptable to users and overcome barriers to behaviour change. The Person-Based Approach can help to optimise interventions to maximise acceptability and engagement. This article presents a novel, efficient and systematic method that can be used as part of the Person-Based Approach to rapidly analyse data from development studies to inform intervention modifications. We describe how we used this approach to optimise a digital intervention for patients with hypertension (HOME BP), which aims to implement medication and lifestyle changes to optimise blood pressure control.

**Methods:**

In study 1, hypertensive patients (N = 12) each participated in three think-aloud interviews, providing feedback on a prototype of HOME BP. In study 2 patients (N = 11) used HOME BP for three weeks and were then interviewed about their experiences. Studies 1 and 2 were used to identify detailed changes to the intervention content and potential barriers to engagement with HOME BP. In study 3 (N = 7) we interviewed hypertensive patients who were not interested in using an intervention like HOME BP to identify potential barriers to uptake, which informed modifications to our recruitment materials. Analysis in all three studies involved detailed tabulation of patient data and comparison to our modification criteria.

**Results:**

Studies 1 and 2 indicated that the HOME BP procedures were generally viewed as acceptable and feasible, but also highlighted concerns about monitoring blood pressure correctly at home and making medication changes remotely. Patients in study 3 had additional concerns about the safety and security of the intervention. Modifications improved the acceptability of the intervention and recruitment materials.

**Conclusions:**

This paper provides a detailed illustration of how to use the Person-Based Approach to refine a digital intervention for hypertension. The novel, efficient approach to analysis and criteria for deciding when to implement intervention modifications described here may be useful to others developing interventions.

## Introduction

This paper presents a novel, efficient way of analysing qualitative data, which can be used as part of the Person-Based Approach [1] to inform intervention development. It also presents criteria for deciding whether to implement a potential intervention modification. The Person-Based Approach [1] illustrated here takes a middle road between participatory [2] and theory- [3] and evidence-based [4] approaches to intervention development, by prioritising and incorporating user perspectives wherever possible, while ensuring the intervention retains all the elements that theory and evidence suggest will be effective in supporting behaviour change. This paper describes how this approach was used to optimise the HOME BP intervention for management of hypertension.

High blood pressure (BP) is the leading risk factor for global disease burden [5], but UK treatment of hypertension remains suboptimal, despite the existence of a range of effective interventions [6]. Reasons for this include clinicians not responding to raised BP readings by intensifying treatment [7] and patients not adhering to medication or lifestyle changes [8].

The TASMIN studies have shown that patients self-monitoring BP at home and implementing pre-agreed medication changes in the face of sustained raised BP readings is a highly effective way of managing hypertension [9,10]. Both TASMIN studies provided face-to-face training for patients in these procedures, but online delivery could provide a cost-effective way of automating the intervention procedures, enabling this intervention to be rolled out more widely. The TASMIN procedures were recently adapted for online delivery with a digital intervention named HOME BP [11]. HOME BP also includes support for making lifestyle changes (e.g. diet, physical activity), since such changes are recommended for reducing hypertension [12]. The planning of HOME BP is reported elsewhere [13].

Within face-to-face consultations, the healthcare practitioner quickly notices if patients do not understand or have concerns about particular procedures, enabling them to immediately respond to concerns and overcome barriers to engagement. This is not possible with digital interventions that are accessed by patients at home. Best practice guidelines therefore recommend eliciting target users’ perceptions of a prototype website, which can enable modifications to the intervention to overcome potential problems with engagement [14, 1, 15]. The Person-Based Approach is a method for optimising digital intervention materials to ensure that they are as acceptable, engaging, persuasive and feasible to carry out as possible, to promote engagement and ultimately effectiveness and implementation [1]. It could be considered a particular approach to user-centred design, with a greater emphasis on behavioural analysis and in-depth qualitative research than is typical of agile user-centred and participatory methods (see [1] for a full discussion of how the Person-Based Approach compares to other approaches). The Person-Based Approach involves collecting comprehensive feedback from target users on a prototype intervention. It is then necessary to undertake an analysis of this feedback that attempts to accommodate user perspectives (which often differ) without removing features of the intervention that theory or evidence suggest may be critical to its success. Often at this point researchers conduct traditional qualitative analyses (such as thematic analysis; [16]), which take considerable time to complete. However, a full scale thematic (or other traditional) analysis at this stage can hold up intervention development. The aim of analysis during intervention development is to iteratively modify the intervention based on user feedback until further qualitative data collection does not identify any significant further modifications that can and should be made–a specific form of data ‘saturation’. To do this, it is necessary to rapidly and efficiently analyse feedback as soon as it is collected, so that modifications can be made before the next wave of data collection. In this paper we illustrate a systematic, yet pragmatic solution to analysing qualitative data from development studies, which we have used successfully to develop multiple interventions. The analyses undertaken for this purpose are necessarily quite different from more traditional qualitative methods, such as thematic analysis [16] (which we often conduct in parallel, or after development, to enable us to also obtain insights from our data that are less specific to the intervention).We also present criteria for deciding when to implement an intervention modification. We believe that the approach illustrated here will be of use to others developing complex interventions.

## Methods

### Design

The development phase of HOME BP involved three qualitative interview studies. Study 1 involved think-aloud interviews [17], where patients viewed HOME BP with a researcher whilst saying what they were thinking out loud. In Study 2 patients used HOME BP alone for 3 weeks and were then interviewed about their experiences of using the intervention. Both of these studies sought to explore patients’ perceptions of HOME BP in order to identify areas of the intervention which might require modification in order to maximise engagement. In Study 3 we purposively recruited participants who *did not* want to use HOME BP, to explore their perceptions of the proposed intervention, using semi-structured telephone interviews.

Ethical approval was gained from NHS NRES Committee London—Fulham research ethics committee (ref: 13/LO/1502) and the University of Southampton ethics committee (ref: 7251).

#### HOME BP for patients

HOME BP is a web-based digital intervention which facilitates home monitoring of BP, medication changes (if patients’ BP remains above target) and lifestyle changes (salt restriction, a DASH-inspired diet [11], physical activity, weight-loss and alcohol reduction). Lifestyle changes were considered an optional component, with the main focus being on home monitoring and making medication changes, since medication is the most effective means to control BP [12] and patients might not be motivated to make lifestyle changes [18]. The HOME BP website is described in detail elsewhere [11], but a brief overview is provided in Table 1.

**Table 1:** An overview of the HOME BP website.

Content	Description
Session 1- An introduction to HOME BP	This session contains: • An interactive quiz about the health benefits of taking the correct medication. • Rationale for monitoring BP at home for one week every month (i.e. overcoming clinical inertia and providing a reliable measure of BP) • Discussion of common concerns (e.g. side effects of changing medication). • Information about the prescriber and supporter’s role in HOME BP.
Session 2- Learning to monitor BP at home	This session contains: • A video and instructions showing patients how to monitor their BP at home. • An explanation of what BP readings mean using a traffic light system (red = very high, amber = too high, green = normal, blue = too low). • A description of what to do if you have very high (red) or very low (blue) readings when monitoring in the study. • Patient stories about using HOME BP successfully to control BP.
Monthly home monitoring	Once patients have completed session 2 they are able to monitor their BP for 7 consecutive (if possible) days a month. They can then enter their readings into HOME BP to receive personalised feedback about whether the average of their readings means that they require a medication change. If patients require a medication change then their prescriber is informed and patients can write a message within HOME BP to the prescriber about their readings (e.g. if there are any factors that the patient wants the prescriber to consider when prescribing).
Session 3- An introduction to lifestyle changes that can control BP	This session contains: • An explanation of how lifestyle changes can help to control BP. • Information about the benefits of each lifestyle change that HOME BP can support (diet, physical activity, weight loss, salt restriction and alcohol reduction). • Information about the ease of making changes, to increase confidence. • The ability to choose which lifestyle change(s) to try.

The full HOME BP intervention includes support from a “prescriber” (a GP or nurse prescriber) who agrees potential medication changes with the patient at baseline, which are implemented later by sending the patient a prescription if their BP readings (entered into HOME BP) remain above target for two consecutive months [11]. The patient can also meet with a “supporter” (a nurse or healthcare assistant) if they have any problems with monitoring their BP at home, or if they want to discuss the lifestyle changes that they wish to implement. During the studies reported here, patients only viewed the three core sessions of the HOME BP website, and did not have support from a prescriber or supporter as in the full HOME BP intervention.

### Recruitment and procedure

#### Studies 1 and 2

Patients in study 1 and 2 were recruited through Primary Care from practices in the South of England. Eligible patients were aged 18 or over, with essential hypertension, prescribed at least one medication for hypertension and were free from terminal disease and any other health condition which would prevent self-monitoring (e.g. dementia). Eligible patients received an invitation letter to take part in the study, along with a participant information sheet and reply slip. Interested patients returned the reply slip or phoned or emailed the researchers to express an interest in the study.

In study 1 twelve patients participated in three think-aloud interviews (N = 36 interviews), which each involved viewing a different HOME BP session with a researcher (KM a senior research assistant or AvW a postgraduate student, who were given detailed training). Consent was taken at the beginning of the first interview. Patients were asked to say everything that they were thinking out loud whilst they viewed HOME BP. A short semi-structured interview followed, which explored patients’ beliefs about managing their hypertension, what they liked and disliked about the intervention content they had viewed, and the costs and benefits of taking part in the behavioural change presented in each session. It took three months to complete the data collection and analysis for study 1, this was an iterative process which involved conducting a few interviews (approx. 1–5), then analysing the data and modifying HOME BP (this took around a week), before collecting more data.

Eleven patients took part in study 2, each returned a completed consent form by post. After this they were posted a BP monitor to use and given access to the online HOME BP programme which they used alone for approximately three weeks. Patients were asked to complete all three sessions described in Table 1, and to complete and record online, a week of home BP readings. Patients were provided with a diary to keep notes on what they thought of each session. After this they took part in a semi-structured telephone interview (with KM or RB, an experienced qualitative researcher) which asked participants how they found using the intervention at home and about any concerns, costs or benefits that they could see to using the intervention. Prior to each interview the researcher looked at what the patient had viewed online to check the validity of reported usage and to inform the conversation, all self-reported usage matched objective usage data. The process for study 2 took approximately 4 months. Interviews were analysed in batches of 1–5 as in study 1.

Studies 1 and 2 provided different insights. The think-aloud interviews in study 1 were conducted with a researcher present and provided very detailed, immediate, observable reactions to everything that the participant saw in the intervention, enabling crucial changes to be made to the detail of content, e.g. to nuances in arguments to improve persuasion, which are often less easily picked up within longitudinal qualitative work like that employed in study 2. The longitudinal qualitative work in study 2 provided insight into how the participant would use the intervention when they were alone, this approach can provide additional insight into any problems with navigation or with trying out behavioural changes.

#### Study 3

In studies 1 and 2, we found very few people were not interested in using HOME BP to perform the main target behaviours (monitor BP or make medication changes). We therefore sought to purposively sample further individuals who would not want to use a website like HOME BP (Study 3). BP UK advertised this third study to members with hypertension. The advert explained the behavioural changes involved in HOME BP and invited participation from people who would not like to use such a programme to make these changes. Patients who expressed interest were sent a consent form, which was completed and returned to the researchers. Seven participants took part in a semi-structured telephone interview (with a medical student who was given detailed training). During this interview, the student explained what was involved in the HOME BP procedures (taking BP readings at home with readings emailed to a GP, making medication changes remotely, and making lifestyle changes) and asked participants what they liked and disliked about each procedure in turn. Participants’ perceptions of the costs and benefits of each change were also explored.

We had quite limited time to conduct study 3 (one month to conduct interviews, analysis and modifications). Our advert appeared to work well, so if we had been able to spend further time recruiting patients from other sources (e.g. other blood pressure related charities or Primary Care) then we would likely have been able to recruit more participants. We would recommend that future studies trying to sample the views of people who might not want to use an intervention take a similar approach to the advert we used (described above), but if more time is available advertise in multiple places to maximise uptake.

Participants in all three studies were each given a £10 gift voucher for each interview they participated in. A description of the demographics of participants who took part in all three studies can be found in Table 2. As negative feedback is especially useful for optimising digital interventions and recruitment materials we deliberately elicited this across all three studies, whilst at the same time looking out for positive feedback that might indicate that intervention components are liked by most users despite some negative reactions.

**Table 2:** Participant characteristics.

Characteristic	Study 1 (n = 12)	Study 2 (n = 11)	Study 3 (n = 7)
Age: Median (range)	69 (51–81)	69 (41, 83)	65 (47–76)
Gender	6 female (50%)	7 female (63%)	3 female (42%)
Years since diagnosis: Median (range)	8 (0.5–26)	20 (3–33)	10 (1–20)
Education level *No formal*			
	0	2 (18%)	1 (14%)
*GCSE (or equivalent)*	0	1 (9%)	
*A level (or equivalent)*	6 (50%)	2 (18%)	2 (29%)
*Diploma*	4 (33%)	1 (9%)	2 (29%)
*Degree*	2 (17%)	4 (36%)	2 (29%)
*Not stated*		1 (9%)	
Ethnicity			
*White British*	11 (92%)	10 (91%)	7 (100%)
*Indian*	1 (8%)		
*Chinese/South East Asian*		1 (9%)	
Employment			
*Full time*	2 (17%)	3 (27%)	
*Self-employed part time*	1 (8%)		
*Retired*	9 (75%)	7 (64%)	5 (71%)
*Unemployed*			2 (29%)
*Not stated*		1 (9%)	

### Data analysis

All data from the interviews were transcribed verbatim. The analysis for study 1 and 2 identified potential barriers to engagement with HOME BP and the behavioural changes involved (self-monitoring, medication changes, lifestyle changes). Interview feedback was used to identify modifications that might optimise the intervention, to make it as acceptable, persuasive, motivating, engaging and feasible to implement as possible. We took an iterative approach, moving between data collection, analysis (identifying modifications which might be needed), modifying the intervention and then further data collection. Saturation was deemed achieved in studies 1 and 2 when participants raised no new concerns in later interviews.

The analysis was carried out by KB (an experienced intervention developer and qualitative researcher) and KM (a senior research assistant). Firstly the researchers familiarised themselves with the data by reading and re-reading the transcripts. The researchers then worked line-by-line through each transcript tabulating all aspects of the data which showed positive perceptions of the intervention or potential barriers to engagement with HOME BP and the behavioural changes involved. For each potential barrier recorded in this table, the research team considered whether a modification to HOME BP could suitably overcome the barrier. If so then a solution was recorded in this table, discussed with LY (an experienced intervention developer) and the modification was implemented.

The criteria used for deciding whether to make a modification to the intervention are shown in Table 3. Modifications were made if they were likely to impact on behaviour change or a precursor to behaviour change (e.g. acceptability, feasibility, persuasiveness, motivation, engagement) and were prioritised based on the MoSCoW (Must have, Should have, Could have, Would like) criteria [14] (see Table 3). Modifications were made in line with the Person-Based Approach’s common guiding principles [1] and the Guiding Principles for the intervention (see Table 3). Modifications that were very uncontroversial and easy to conduct, (e.g. clarifications), were implemented immediately. In some cases it was obvious that a potential change was high priority even if only one person said it, as it would likely impact on behaviour change (or a precursor to behaviour change) for other people. In other cases more data were collected to seek further views before implementing a change. An example of our tabulation of the interview data can be seen in Table 4.

**Table 3:** Criteria for making modifications.

Criteria	Means
*Criteria for deciding whether to make modifications*	
Important for behaviour change	The modification is likely to impact behaviour change or a precursor to behaviour change (e.g. acceptability, feasibility, persuasiveness, motivation, engagement).
Consistent with Guiding Principles	The modification is in line with guiding principles of the intervention. Guiding principles incorporate theory, evidence and user perspectives to provide principles which summarise what an intervention needs to provide in order to achieve its aims, which guide intervention development [1]. For example one of the HOME BP guiding principles was that the intervention should motivate patients and practice staff to undertake medication titration, should facilitate this titration and should be low cost and easy to implement [13].
Consistent with Common Guiding Principles	The modification is in line with common guiding principles [1]: to support autonomy, promote competence, and provide a positive emotional experience and sense of relatedness
Uncontroversial and easy	An uncontroversial and easy to implement solution that doesn’t involve major design changes, e.g. simplifying or clarifying a sentence that was misunderstood. These changes were implemented straightaway.
Repeated by several participants	This point was made by more than one participant.
*Criteria for prioritising which modifications to make (MoSCoW)*	
Must have	This modification must be made in order for the intervention to be effective in changing a participant’s behaviour (given what we know about the evidence base).
Should have	This modification should be made if possible as it may impact effectiveness, but may be able to be delivered in a different way, or is in some way less critical than a Must have.
Could have	This modification would be useful, but may be less critical to behaviour change than a ‘should have’ and may only be implemented if time and resources are available.
Would like	This modification is not needed to support behaviour change, but could be useful if time and resources allow.

**Table 4:** Example of table of iterative intervention changes made during analysis.

Page or aspect of the intervention	Positive comment	Negative comments	Suggested change	Reason for change	Priority (MoSCoW)	Agreed change (if no change agreed, explain why)
Session 1: Page “What do I do next to get started?” Session 2: Page: “Monitoring your BP at home” Session 2: Page: Recording your readings”		Confusion over how often to monitor blood pressure, repeated by several participant, e.g. “So am I supposed to monitor my BP every day?”	Instructions made clearer to state monitor your blood pressure once a day for a week. This instruction needs to be repeated in sessions 1 and 2 as participants are forgetting between sessions.	**Important to behaviour change** **Guiding Principles** (home monitoring crucial to facilitate titration) **Common Guiding Principles** (promote *competence* in following procedure) **Uncontroversial and Easy** **Repeatedly**	**Must have**- crucial to following the self-monitoring procedure	Agreed.

Analysis of the interview data from study 3 was also undertaken using the same methods as in studies 1 and 2 (carried out by RG, a research fellow and KB). Here the focus was on exploring patient’s perceptions of hypothetically using an intervention like HOME BP and the behavioural changes involved (self-monitoring, medication changes, lifestyle changes). Potential barriers to uptake were identified and considered in detail as to which should be addressed in recruitment materials to overcome barriers and maximise uptake.

## Results

Studies 1 and 2 are presented first, followed by study 3. The findings are organised and presented under each of the behavioural changes involved in HOME BP. The modifications that were implemented to optimise the intervention and recruitment materials are described and an overview of these is provided in Table 5. Quotes are provided and the following labels are used to show which study the participant quotes are sourced from: S1 (study 1), S2 (study 2), and S3 (study 3).

**Table 5:** Changes made to HOME BP based on patient feedback.

	Interview feedback	Changes made to HOME BP
1	Some patients were confused by the instructions about how often to monitor their BP, assuming that they needed to monitor every day, rather than for one week every month. Some patients who had seen an explanation of this in session 1 forgot by the following week when they looked at session 2.	Instructions of when to monitor BP were rephrased to make them clearer, and repeated in several places to reinforce them for patients who might forget between sessions.
2	Concern that home BP readings were unreliable because of fluctuations in blood pressure.	We added an explanation that BP does naturally vary, which is why monitoring for 7 days and then taking the average of these readings (which HOME BP does) is an accurate way of measuring blood pressure.
3	A few participants did not want to tighten the arm cuff as they found it uncomfortable when the cuff tightened during BP measurement.	A sentence was added to the blood pressure monitoring instructions to explain the necessity of tightening the arm cuff, in order to avoid measurement errors.
4	Anxiety about monitoring blood pressure correctly at home.	We changed the blood pressure monitoring procedure to include one week of practice monitoring. Patients were given the option of sending practice readings to a practice nurse who could check them and provide support as needed, or recommend a further week of practicing monitoring if readings appeared potentially inaccurate (e.g. unusually variable).
5	Patients did not believe that their GP would exhibit clinical inertia (i.e. not prescribe medication quickly enough when it was required).	We reframed the rationale for home monitoring. Instead of telling patients about clinical inertia, we instead sympathised with GPs’ perspective, explaining that GPs find it hard to know whether to change a patient’s medication based on one-off clinic readings which may be inaccurate, but that home monitoring provides GPs with more robust evidence on which to base prescribing decisions.
6	A few patients felt anxious about the potential for health problems after viewing the quiz in session 1.	A message was added to the end of the quiz which reinforced that taking the right medication could reduce the risk of these health problems.
7	A few patients wanted to meet with their prescriber if they needed to make a medication change, rather than doing this remotely.	We added an explanation to HOME BP that the prescriber was going to implement the medication that the patient and prescriber had agreed on at the baseline medication review, to remind the patient that they had already discussed this medication change.
8	Patients did not understand that salt can be hidden in foods.	An explanation of this was added into the third session of HOME BP.
9	Some patients questioned the link between blood pressure and dementia or kidney problems.	Explanations of how raised blood pressure increases the risk of dementia or kidney problems were added to HOME BP, which appeared to be acceptable to patients.
10	Some patients in study 3 were concerned that the website and not their prescriber would be responsible for deciding when a medication change was necessary. These patients felt that a website would not understand all factors which could be involved in raised BP.	We were able to address these concerns in our patient invitation letter and participant information sheet for the HOME BP trial by providing reassurance that their prescriber, rather than the website would make decisions about medication changes based on their personal needs (e.g. we included “HOME BP will let you and your GP know if your blood pressure is too high. The GP can then decide if you need extra or different medications to help lower your blood pressure.” to our participant information sheet)
11	One participant in study 3 was concerned about security of the HOME BP website, since his email had been hacked.	We ensured that our participant information sheet stated that HOME BP is secure.

### Studies 1 and 2

Studies 1 and 2 explored participants’ perceptions of HOME BP, identifying potential barriers to engagement with the intervention.

#### Monitoring BP at home

Often participants reported that HOME BP would empower them to self-manage their hypertension by monitoring their BP at home, enabling them to take more control of their health. Some participants were pleased to learn how to correctly self-monitor their BP. Many participants reported benefits of home monitoring, such as eliminating the wait for a GP appointment, or yielding more accurate readings than going to the GP.

In the early study 1 interviews, a few participants found the online instructions regarding how often they should monitor their BP confusing. Changes were made to improve the clarity of these instructions (see Table 5, point 1), which seemed successful as reports of confusion diminished after this change. Some participants were also concerned that readings were unreliable because they fluctuated. Explanations were therefore added regarding how HOME BP overcomes this problem (Table 5, point 2). One participant in study 2 did not seem to be doing her arm cuff up sufficiently to accurately measure her BP, as this felt uncomfortable, so the instructions about this were modified (Table 5, point 3).

Some participants in study 1 and 2 were anxious about monitoring their BP correctly and one participant in study 1 deemed it important for their prescriber to take a reading in addition to their home readings to check accuracy.

“I was quite worried about the reading and I think I may have been doing it wrong, which was causing my BP to be higher.”(S2-P1)

“I still think you should check somebody to make sure that it was within the amber zone, because if there was a mistake or something wrong with the machine or whatever, I think you should do something.” (S1-P10).

In response, we made changes to the BP monitoring procedure to include a practice week which could help increase participants’ confidence (Table 5, point 4).

Participants in study 2 were able to monitor their blood pressure at home for a week. Many of these participants expressed positive feelings such as reassurance and a sense of achievement when their readings were within a normal range. These participants also noted that they might worry initially if their readings were high, but that at least this way something could be done about it.

“It said 'Congratulations, you're normal.' So that was great. (Laugh).” (S2-P2)

“If you had a high reading and it came back red then you would obviously feel possibly a bit anxious but at least you'd found out…and it could be dealt with.”(S2-P8)

Whilst the majority of participants anticipated self-monitoring would be easy, a few participants in both studies 1 and 2, particularly those working full time, felt they might not have time to self-monitor regularly.

#### Making medication changes

In the initial prototype of HOME BP used in study 1 participants were told about the clinical inertia surrounding prescribing medication for hypertension and explained how the HOME BP procedures could help overcome this, to ensure that participants get the best treatment possible. However, some participants did not believe that their GP might exhibit clinical inertia, arguing that *their GP* must be the exception.

“I don’t particularly think my GP is in that situation…I think that if someone had a GP who wasn’t as caring it would be helpful. I have to say I’m one of the few remaining people in the world who still has a family GP.”(S1-P3)

At this point it was clear that the rationale for home monitoring needed to be reframed to ensure it was consistent with, rather than a challenge to, peoples’ trust in their GPs (Table 5, point 5). Participants interviewed after this change appeared convinced by the new rationale and wanted to engage with the HOME BP procedures.

“This would help me and him (GP). Cos I could give him the heads up, we should do something about it. So that’s, that’s good stuff yea. You’re convincing me that it would be a good idea.”(S1-P4)

Although not all participants believed that their GP would exhibit clinical inertia, they did acknowledge that GPs often have very limited time. Many saw HOME BP as a tool which could save GPs time and thus facilitate optimal prescribing.

“You can go in loaded with evidence, ‘I’ve been doin this and ‘I’m on the BP study’ and if they’re aware of it they’ll go ‘ah, better listen to this bloke’. I’ve collated, then its goin to save the doctor time and save me a lot of trouble as well. And improve me, which is important.”(S1-P9)

However, a small minority felt that this would come at the cost of providing a lower quality service and wanted to meet with their prescriber if they needed a medication change rather than make medication changes remotely based on advice from HOME BP.

“I like to ask about any potential side-effects … And have a face-to-face discussion of that with a doctor if the programme itself tells me that I need to take more then I might feel that I wanted to speak to someone about it first.” (S2-P5)

“I think to be prescribed medication via computers without actually seeing the GP makes it very impersonal …I think its downgrading.”(S1-P12)

Since implementing pre-agreed medication changes is likely an important mechanism in overcoming clinical inertia [9,10], and is also necessary to maximise cost-effectiveness and feasibility (by reducing the need for consultations), we decided not to make this change to procedure. We suspected that this concern would be reduced by the face-to-face baseline medication review with the patient’s prescriber, where future medication changes are discussed and agreed (this medication review occurs in the full HOME BP intervention, but for pragmatic reasons could not implemented in studies 1 and 2). We therefore added reassurance that prescribers were going to implement medication changes which had been agreed with the patient at baseline to the feedback that patients get when entering BP readings into HOME BP and added a facility for patients to communicate any concerns to prescribers (e.g. about side-effects) through HOME BP (Table 5, point 7).

Most participants viewed the quiz in session 1 about the benefits of taking the correct medication for their hypertension very positively. A few participants in study 1 reported feeling anxious after viewing the quiz, as they did not want to get the health problems that were mentioned. As raising levels of concern without promoting self-efficacy might trigger denial, rather than increased motivation [19], we decided to add a message to the end of the quiz which reinforced that people could reduce the risk of these health problems by taking the correct medication (see Table 5, point 6). No further participants reported feeling anxious after this.

Many participants were happy to make medication changes if they were needed, in order to avoid health problems. Some participants expressed a preference for making medication changes rather than lifestyle changes as less effort was required.

*“Medication is a quick fix*. *I understand it’s like a band aid on a shrapnel wound*, *but it is a quick fix because doing (lifestyle changes)* … *I mean*, *no idiot would say not to do it*, *I mean*, *but the idiots won’t*, *like myself*.*”* (S1-P10).

Some participants were concerned about making medication changes because they felt their BP was well controlled by their current medication or they were concerned about potential side-effects. However, many conceded that they would make a medication change if one was needed.

“I can’t sit about at home all day feeling poorly ‘cause I’ve had to change (medication). If it was life threatening, then you’d think twice about it…which I suppose in some ways it could be with BP anyway, but um. Um I suppose really, if, if I had to have it changed, I’d have to just say well I give myself a fortnight to get used to these new um this new medication and just see how it goes.”(S1-P6)

One participant stated at the start of study 1 that she would never take the medication she had been prescribed for high BP. However, by her third interview viewing HOME BP she decided to start taking her medication.

“Obviously I’ve got to do something. BP rises as you get older …I don’t want to take the medication that’s been offered, but um I’ve given in.”(S1-P3)

#### Making lifestyle changes

A few participants in study 1 and 2 commented that HOME BP had raised their motivation for making lifestyle changes.

“(The Lifestyle changes) would make me feel like I’m taking control rather than like I said at the first, I feel that I’m not in control, basically. But by choosing to do this, I’m, I’m taking control. I am gonna do my extra exercise… 5 days a week or whatever. I’m gonna do this because I’m gonna take control. That’s quite a different mind-set to the mind-set I had at the start. In fact, that’s a complete reverse of the mind-set that I had at the start.”(S1-P3)

When asked, all participants could report some benefits of making lifestyle changes and for a few, health benefits seemed to be a sufficient motivator. However, most described reasons why they personally would struggle to make lifestyle changes. Reported barriers to increasing physical activity included existing physical health problems, ageing, insecurities about physical appearance when being active and a lack of time or money.

“I would be concerned about the physical action, because I suppose as you get older and there’s wear and tear on your bones and you might have a few aches and pains.”(S2-P3)

Participants also did not appear particularly motivated to make changes to their diet, often believing that they were already eating healthily. For instance, some believed that as they were not adding salt to their diet, they were eating a low salt diet. However, it appeared from the foods that they talked about eating that they might not know about salt which is contained in processed foods, so an explanation about hidden salts was added (Table 5, point 8).

Part of participants’ lack of motivation seemed to be due to distrust in the health benefits of lifestyle changes or a lack of confidence in their ability to make such changes.

“Salt, I don’t know, maybe that comes in trends like sugar. I don’t know enough about that to actually say if that would be beneficial to you or not.”(S2-P3)

“I resemble my mother very much, um and I remember that her idea of healthy living was to go out for a good walk and she died. She went mad, went potty. Demented at 50 something. And she lingered for a few years and died at 72 I think. So I really don’t believe all this business of going for a good walk”(S1-P2)

“The weight loss one would be nice but …I haven’t got the determination I don’t think.”(S2-P9)

Some participants questioned the link between BP and dementia or kidney problems (Table 5, point 9 shows how we responded to this).

All participants were positive about the prospect of having a Supporter who they could talk to about lifestyle changes or home monitoring if they wanted to.

### Study 3

Participants in study 3 had responded to an advert saying they did not want to use a BP management website. We explored their views of using an intervention like HOME BP, and the behavioural changes involved, to identify potential barriers to uptake and how these could be addressed via our recruitment materials. Below we present participants’ perceptions of making medication changes as well as factors identified as unique to study 3 participants, which were not observed in studies 1 and 2. Study 3 participants’ views on self-monitoring BP at home and making lifestyle changes were consistent with participants in study 1 and 2, so are not shown here.

#### Making medication changes

Most participants in study 3 had some concerns about making medication changes remotely. Often they anticipated that the website was going to decide on whether they received a medication change, without the involvement of their GP. They perceived this as dangerous because the website would not understand other factors which might be responsible for raised BP.

*“I’m not sure about entering into a website… my GP knows my other medical problems that do impinge on what can be done about my BP*. *Some anonymous website is not going to know what I feel*.*”*(S3-P5)

“If your father dies or something. The program doesn’t pick that up but the doctor could… going to the website to do it, you’re actually making things worse.”(S3-P4)

In HOME BP, prescribers (rather than the website) still make a final decision about whether to implement a medication change. There is a dedicated space within HOME BP for people to record any issues they would like the practitioner to consider when prescribing. To overcome this potential barrier to uptake we ensured that we noted in the recruitment materials that medication changes would be decided by the GP and not the website (Table 5, point 10).

#### Additional factors that were unique to study 3 participants

In study 3 one participant was concerned that using the internet to send BP readings to inform medication changes might be a security risk, since his email had been hacked (Table 5, point 11 shows how we responded to this). Two other participants were less computer literate and identified using a computer as something that other people do, so would likely not have been persuaded to use an intervention like HOME BP, even with modifications to the recruitment materials.

Whilst patients in study 1 placed a lot of trust in their GPs, not believing arguments about clinical inertia, in contrast, a few participants in study 3 exhibited distrust of their GPs. These participants argued that GPs do not understand how to treat hypertension, or that GPs only prescribed medication because they are financially incentivised to do so, rather than to protect patients’ health.

“All he’s doing is monitoring it because he gets money for monitoring it … he says you’ve got BP try this if it doesn’t work try another thing. And there’s no method or madness behind it. He’s alright on other things but BP I think is a waste of time, they don’t know what they’re doing.”(S3-P4)

“They have got targets to reach, and sometimes these are financial targets. And sometimes I feel like I’m being sold a product.”(S3-P3)

One participant in study 3 did not believe that GPs would bother to look at BP readings unless the participant was present, so disliked the idea of the GP being sent his readings.

## Discussion

Overall, patients in studies 1 and 2 indicated that the HOME BP intervention appeared acceptable, persuasive, motivating and engaging. However, patients also had some important concerns which could create barriers to engagement, and patients in study 3 highlighted potential barriers to uptake of HOME BP. This paper has presented a novel, efficient and systematic method that can be used to rapidly analyse qualitative development study data, presenting how we were able to use participant feedback to modify the intervention and recruitment materials to improve acceptability, credibility and persuasiveness. Below our findings are discussed in relation to the wider literature.

### Relating findings to the wider literature

Patients in all three studies generally believed self-monitoring BP at home to be an empowering, easy and beneficial process. Interviews highlighted two barriers to self-monitoring which were useful to address: first, concern about the accuracy of fluctuating readings, which has also been observed elsewhere [20]; second, concern about performing monitoring correctly, which prompted the inclusion of a ‘practice’ week of monitoring which patients could ask to be checked by a practice nurse. This addition was not only useful and reassuring for patients, but likely also for practitioners, who are sometimes concerned about the accuracy of home readings [21]. Previous studies indicate that patients can follow home monitoring procedures correctly [22], but raising patients’ confidence with this extra practice should maximise acceptability and engagement.

Many patients were happy to make medication changes, if needed. Even patients who had concerns about changing medications (e.g. fearing side effects) balanced these concerns against the health protection that a medication change could provide, conceding that they would make medication changes if necessary, a phenomena which has been observed elsewhere [23, 24]. Whilst patients appeared motivated by HOME BP and reported a willingness to make medication changes, the wider literature shows a lack of adherence to medication within hypertensive populations [25, 26]. It will be useful to examine how frequently patients implement and adhere to recommended medication changes within the HOME BP trial. Review evidence suggests that home monitoring increases patients’ understanding of their condition and can help people feel more motivated to make medication changes [27] as well as increasing adherence to medication [28]. This suggests that adherence to medication changes might be higher in the context of using an intervention like HOME BP than in the wider hypertensive population.

Whilst most patients appeared open to making medication changes, few appeared motivated to make lifestyle changes, suggesting that our plan to position lifestyle change as an optional component of HOME BP, instead prioritising medication changes, was sensible. This finding is consistent with a systematic review which indicates that hypertensive patients can lack motivation for lifestyle changes and perceive difficulties in sustaining such changes [18].

Patients in study 1 and 2 tended to have high levels of trust in their GPs, meaning that our original intervention rationale which mentioned clinical inertia posed a barrier to engagement, which we had not anticipated, but which was simple to address. A few of these patients worried about making medication changes remotely based on advice from a website, rather than meeting with their prescriber. A process evaluation of the TASMINH2 study found that a minority of patients still seek an appointment with a healthcare professional when asked to make medication changes remotely [22], but the TASMINH2 study showed that the intervention was effective and cost-effective, even though this minority did not follow the procedures entirely [9].

Study 3 participants, who did not want to use a website like HOME BP, often reported little trust in their GPs’ management of their hypertension, whilst at other points in the interview arguing that they would rather have their GP, than a website, make decisions about their hypertension medication. It is possible that patients’ trust in digital interventions like HOME BP, which partly replace face-to-face relationships with practitioners, might be influenced by patients’ initial trust in their practitioner. If initial trust in their doctor’s ability to manage a health condition is low then trust in a digital solution may also be low, in line with findings from Andreassen et al [29].

### Implications for intervention development and uptake

This article has demonstrated a novel, efficient way of analysing qualitative data to inform intervention development and criteria for deciding whether to implement a potential intervention modification, both of which will likely be useful to those who develop interventions. Employing a Person-Based Approach [1] to intervention development in this study, using iterative data collection, rapid analysis and predefined criteria for deciding which modifications to implement, enabled us to identify and seemingly overcome potential barriers to patient engagement with HOME BP, increasing the acceptability and persuasiveness of this intervention. Our prior behavioural analyses based on the existing research and theory did not enable us to anticipate all the barriers to engagement which were identified here [13]. This is the reason that the Person-Based Approach uses inductive and iterative qualitative research methods during intervention development, to enable intervention optimisation through modifications to a prototype prior to implementation [1].

The three different study designs employed each provided unique and useful insights. Study 1 provided insight into the details of content which required modification. Whereas study 2, enabled exploration of how participants would find using the website and trying out behavioural changes alone, which highlighted a problem with using the monitoring equipment (which we were then able to address) and confirmed that generally participants were able to carry out self-monitoring alone successfully. Purposively sampling individuals who would not want to use HOME BP (in study 3) was useful as it enabled us to identify incorrect assumptions that patients had made about HOME BP procedures which might influence uptake, which we could then address in our recruitment materials to maximise recruitment. Whilst other studies have tried to map factors which influence trust in e-health [30] these are mainly factors which influence trust once someone has accessed a website, e.g. look and feel. Studies exploring reasons for non-participation in digital interventions are rare, usually relying on non-participation response forms or healthcare practitioners’ opinions [31], rather than in-depth interviews with patients themselves. Such studies suggest that lengthy recruitment materials may be off-putting, or that the intervention was unwanted, but provide little further detail [31]. Exploring the views of those who may decline to participate in advance provides richer detail and can identify changes which may help maximise uptake of digital interventions. Further research would be useful to employ the Person-Based Approach to refine digital intervention recruitment materials and to test whether materials refined in this way lead to better uptake than unrefined materials.

### Limitations

Whilst we were able to explore patients’ perceptions and experiences of using the HOME BP website, we were unable to explore their experiences of implementing medication changes, attending baseline medication reviews with a prescriber, or attending appointments with their supporter. A vital next step to assessing the acceptability and feasibility of HOME BP will be to explore these issues within qualitative processes interviews nested within the HOME BP trial [32]. Two of the interviewers involved in these studies (KM and RB) were involved in the development of HOME BP. Although patients were not told this, it is possible that some might have assumed this to be the case and provided socially desirable answers. However, this seemed less likely, since patients were willing to express negative views (e.g. lack of interest in making lifestyle changes). Studies 1 and 2 both reached our criteria for data saturation, but study 3 did not; it would have been useful to identify more people who did not want to use an intervention like HOME BP. In practice these participants were hard to find and sampling further individuals was not possible within our time constraints, but the data provided by the seven participants was nevertheless extremely useful. Whilst the samples included a fairly even spread of genders and a range of ages, people with lower education levels were under represented in study 1 and people of a non-white British background were under-represented across all three studies. Many of the participants in all three studies were retired and we were able to recruit fewer participants in employment, this is perhaps unsurprising given that the majority of people in both our samples and the general population who have hypertension are aged 65 or over (i.e. retirement age) [33]. It would be useful to interview people from a range of education levels, ethnic backgrounds and employment statuses within the process interviews of the HOME BP trial to explore their views of HOME BP and whether perceptions of the intervention differ in the groups which were under-represented here. The methods described in this paper can be applied to developing interventions for any condition, not just hypertension.

## Conclusion

The study provides rich insights into patients’ perceptions of the HOME BP intervention and demonstrates how employing a Person-Based Approach enables potential barriers to engagement and uptake to be overcome through modifications to the intervention and recruitment materials. The paper also presents a systematic and pragmatic method for analysing qualitative data to inform intervention development, as well as criteria which can guide decisions about whether to implement an intervention modification, which may be useful to other intervention developers. A number of potential barriers to engagement were identified within our first two studies, including a lack of confidence in monitoring BP at home accurately and the intervention rationale challenging patients’ trust in their GPs. Barriers to uptake were also identified, including a belief that the website, rather than a practitioner would decide whether medication changes were warranted. Modifications to the intervention and recruitment materials allowed us to address these barriers and improve the acceptability and feasibility of implementing HOME BP in practice and maximise uptake. Further research is now required to explore the effectiveness and cost-effectiveness of HOME BP, as well as to explore patients’ experiences of implementing the full HOME BP procedures [11].
